# ENPP1 Affects Insulin Action and Secretion: Evidences from *In Vitro* Studies

**DOI:** 10.1371/journal.pone.0019462

**Published:** 2011-05-05

**Authors:** Rosa Di Paola, Nunzia Caporarello, Antonella Marucci, Claudia Dimatteo, Claudia Iadicicco, Silvia Del Guerra, Sabrina Prudente, Dora Sudano, Claudia Miele, Cristina Parrino, Salvatore Piro, Francesco Beguinot, Piero Marchetti, Vincenzo Trischitta, Lucia Frittitta

**Affiliations:** 1 Research Unit of Diabetes and Endocrine Diseases, IRCCS “Casa Sollievo della Sofferenza”, San Giovanni Rotondo, Italy; 2 Unit of Endocrinology, Department of Clinical and Molecular Biomedicine, University of Catania Medical School, Garibaldi Hospital, Catania, Italy; 3 Dipartimento di Biologia e Patologia Cellulare e Molecolare and Istituto di Endocrinologia ed Oncologia Sperimentale del CNR, Università degli Studi di Napoli Federico II, Naples, Italy; 4 Department of Endocrinology and Metabolism, University of Pisa, Pisa, Italy; 5 IRCCS “Casa Sollievo della Sofferenza, Mendel Laboratory”, San Giovanni Rotondo, Italy; 6 Unit of Internal Medicine, Department of Clinical and Molecular Biomedicine, University of Catania Medical School, Garibaldi Hospital, Catania, Italy; 7 Department of Experimental Medicine, Sapienza University, Rome, Italy; University of Bremen, Germany

## Abstract

The aim of this study was to deeper investigate the mechanisms through which ENPP1, a negative modulator of insulin receptor (IR) activation, plays a role on insulin signaling, insulin secretion and eventually glucose metabolism. ENPP1 cDNA (carrying either K121 or Q121 variant) was transfected in HepG2 liver-, L6 skeletal muscle- and INS1E beta-cells. Insulin-induced IR-autophosphorylation (HepG2, L6, INS1E), Akt-Ser^473^, ERK1/2-Thr^202^/Tyr^204^ and GSK3-beta Ser^9^ phosphorylation (HepG2, L6), PEPCK mRNA levels (HepG2) and 2-deoxy-*D*-glucose uptake (L6) was studied. GLUT 4 mRNA (L6), insulin secretion and caspase-3 activation (INS1E) were also investigated. Insulin-induced IR-autophosphorylation was decreased in HepG2-K, L6-K, INS1E-K (20%, 52% and 11% reduction vs. untransfected cells) and twice as much in HepG2-Q, L6-Q, INS1E-Q (44%, 92% and 30%). Similar data were obtained with Akt-Ser^473^, ERK1/2-Thr^202^/Tyr^204^ and GSK3-beta Ser^9^ in HepG2 and L6. Insulin-induced reduction of PEPCK mRNA was progressively lower in untransfected, HepG2-K and HepG2-Q cells (65%, 54%, 23%). Insulin-induced glucose uptake in untransfected L6 (60% increase over basal), was totally abolished in L6-K and L6-Q cells. GLUT 4 mRNA was slightly reduced in L6-K and twice as much in L6-Q (13% and 25% reduction vs. untransfected cells). Glucose-induced insulin secretion was 60% reduced in INS1E-K and almost abolished in INS1E-Q. Serum deficiency activated caspase-3 by two, three and four folds in untransfected INS1E, INS1E-K and INS1E-Q. Glyburide-induced insulin secretion was reduced by 50% in isolated human islets from homozygous QQ donors as compared to those from KK and KQ individuals. Our data clearly indicate that ENPP1, especially when the Q121 variant is operating, affects insulin signaling and glucose metabolism in skeletal muscle- and liver-cells and both function and survival of insulin secreting beta-cells, thus representing a strong pathogenic factor predisposing to insulin resistance, defective insulin secretion and glucose metabolism abnormalities.

## Introduction

Type 2 diabetes mellitus is a complex disorders due to the combination of genetic and environmental factors. Impaired insulin action (i.e. insulin resistance) in liver and skeletal muscle as well as reduced pancreatic beta-cell insulin secretion are pathogenic for type 2 diabetes [1]. In the presence of insulin resistance, glucose homeostasis is preserved by compensatory hyperinsulinemia [2], [3], with type 2 diabetes ensuing only when beta-cells fail to secrete sufficient insulin to adequately counteract impaired insulin sensitivity. Besides the established role of abnormal insulin signaling in predisposing to insulin resistance [4], studies in animal models have proposed that insulin signaling is essential also for beta-cell insulin secretion [5], [6], [7], [8]. Along the same line of evidences, human non synonymous genetic variations which affect the insulin signaling pathway [9], [10], [11], [12] and which have been associated with *in vivo* insulin resistance [13], [14], are also able to affect insulin secretion *in vivo* [13], [15], in isolated human islets [8], [16], [17] and in cultured beta-cells [8], [18]. Thus, an intriguing scenario has emerged suggesting that abnormalities impairing insulin signaling play a role on glucose homeostasis not only by affecting glucose metabolism in liver and skeletal muscle, but also by inducing defective insulin secretion [19], [20].

Ectonucleotide pyrophosphatase phosphodiesterase 1 (ENPP1) is a class II transmembrane glycoprotein, which inhibits insulin receptor (IR) signaling and which has been, therefore, proposed as a candidate for insulin resistance [20], [21]. In fact, in both cultured cells [22], [23], [24], [25], [26], [27] and mice [28], [29], increasing ENPP1 expression causes impaired insulin signaling and action. Moreover, ENPP1 is overexpressed in skeletal muscle, adipose tissue, fibroblasts and lymphocytes of insulin-resistant individuals [21], [30], [31], [32], [33]. Further support to the notion that ENPP1 may play a role on human insulin resistance derives from studies on the *ENPP1* K121Q polymorphism [34], which has drawn some attention as a genetic determinant of human insulin resistance. Indeed, the Q121 variant has been associated with insulin resistance in several [35], [36], [37] although not all [38] large studies. Interestingly, it also predicts incident major cardiovascular events [39], an important clinical outcome of insulin resistance. These epidemiological associations have been proposed to be mediated by a stronger inhibitory activity on IR signaling, as compared to that exerted by the K121 variant [25], [34], [40]. However, such functional studies pointing to the K121Q polymorphism as a gain of function aminoacid substitution, have been obtained in non typical insulin target cells [25], [34], [40] and may not, therefore, be considered as conclusive. More recently, a deleterious effect of the Q121 variant on *in vivo* insulin secretion has been reported [37]. Whether this is given by a direct detrimental effect on beta-cells or, in contrast, it is secondary to alterations of the metabolic milieu related to whole body insulin resistance, is an additional open question, which deserves further studies to be answered.

The aim of this study was to deeper investigate *in vitro* the mechanisms through which ENPP1 plays a role on insulin signaling, insulin secretion and eventually glucose metabolism. To this purpose, the effect of ENPP1 expression (either the K121 or the Q121 variant) was investigated in the three most important cell types for maintenance of glucose homeostasis (i.e. liver-, skeletal muscle- and pancreatic beta-cells). In details, we studied i) insulin-induced IR activation in all three cell types, ii) downstream insulin signaling and subsequent insulin action on glucose metabolism in liver- and skeletal muscle-cells and iii) beta-cells insulin secretion and survival. The data we obtained clearly indicate that ENPP1, especially when the Q121 variant is operating, exerts a direct deleterious effect on all these cell types, thus representing a strong candidate as a pathogenic factor predisposing to insulin resistance, defective beta-cell insulin secretion and glucose metabolism abnormalities.

## Results

### Studies on IR autophosphorylation

**Figure 1:**
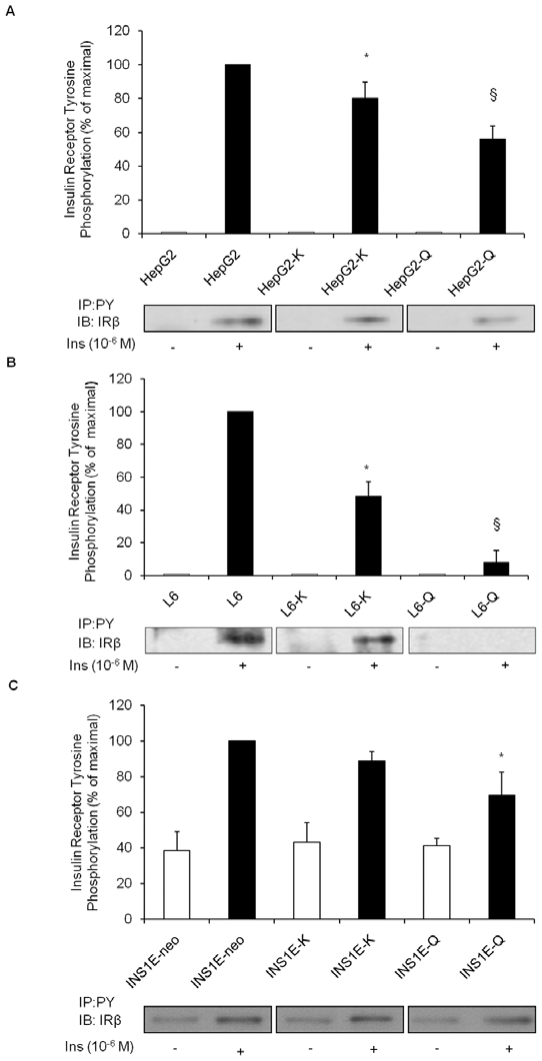
Studies on IR autophosphorylation in HepG2 (A), L6 (B) and INS1E (C) cells. Cells were either transfected, or not, with *ENPP1*-K121 cDNA or with *ENPP1*-Q121 cDNA and then stimulated with insulin as described in methods. Equal amount of protein from cell lysates was immunoprecipitated with anti-PY antibody, separated by SDS-PAGE and probed with IR-beta subunit antibody. Bars (upper panels) represent quantitative analysis of IR autophosphorylation calculated as percentage of that of stimulated untransfected cells in each experiment (mean ± SEM), while representative immunoblots from the same experiment are shown in lower panels. *(* ***A*** *)* In HepG2 cells, insulin induced IR-beta subunit autophosphorylation. This effect was significantly reduced in HepG2-K (*p = 0.02, fourth vs. second bar, n = 3 experiments in separate times) and more profoundly in HepG2-Q (^§^p<0.001, sixth vs. second bar, n = 3 experiments in separate times) cells. When properly tested, a progressive reduction was observed from control to HepG2-K and then HepG2-Q cells (p for trend<0.001). *(* ***B*** *)* In L6 cells, insulin induced IR-beta autophosphorylation. As compared to control cells, insulin effect was significantly reduced in L6-K (*p = 0.01, fourth vs. second bar, n = 3 experiments in separate times) and more profoundly in L6-Q cells (^§^p<0.001, sixth vs. second bar, n = 3 experiments in separate times). When properly tested, a progressive reduction was observed from control to L6-K and then L6-Q cells (p for trend<0.001). *(* ***C*** *)* In INS1E cells, insulin induced IR-beta autophosphorylation. As compared to control cells, insulin effect was only slightly and not significantly reduced in INS1E-K (fourth vs. second bar, n = 3 experiments in separate times) and significantly reduced in INS1E-Q cells (30% reduction *p = 0.02, sixth vs. second bar, n = 3 experiments in separate times). When properly tested, a progressive reduction was observed from INS1E-neo to INS1E-K and then INS1E-Q cells (p for trend = 0.024).

IR tyrosine autophosphorylation was studied in human liver HepG2 cells, rat skeletal muscle L6 cells and rat pancreatic INS1E beta-cells. To this purpose, cells were transfected with either *ENPP1*-K121 (HepG2-K, L6-K and INS1E-K) or *ENPP1*-Q121 (HepG2-Q, L6-Q, and INS1E-Q) cDNA, and then stimulated with insulin as described in methods. Immunoblot analysis showed that insulin stimulation induced autophosphorylation of IR-beta subunit in HepG2 (Figure 1 A), L6 (Figure 1 B) and INS1E-neo (Figure 1 C) control cells. This effect was variably reduced in HepG2-K (20% reduction; Figure 1 A), L6-K (52% reduction; Figure 1 B) and INS1E-K (11% reduction; Figure 1 C). Such reduction was approximately doubled in HepG2-Q (44% reduction; Figure 1 A), L6-Q (92% reduction; Figure 1 B) and INS1E-Q cells (30% reduction; Figure 1 C).

These data, strongly suggest that ENPP1 is an inhibitor of IR autophosphorylation in typical insulin target cells, as well as in insulin secreting beta-cells and that the Q121 variant is a gain of function aminoacid substitution with increased inhibitory activity.

**Figure 2:**
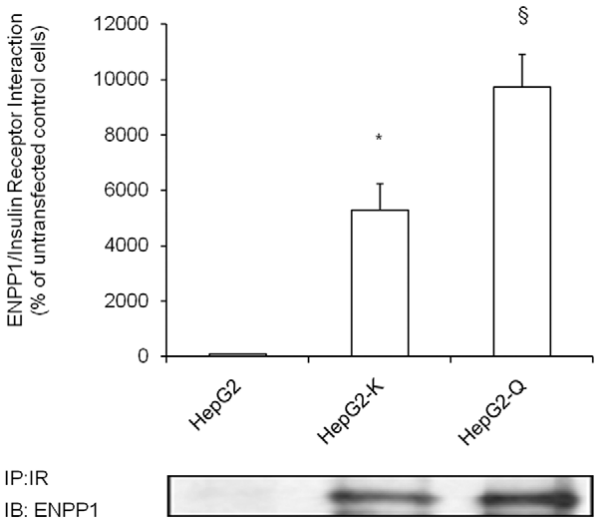
ENPP1/IR interaction. Lower panel: HepG2 cells were either transfected (lanes 2–3) or not (lane 1) with *ENPP1*-K121 cDNA (lane 2) or with *ENPP1*-Q121 cDNA (lane 3) and stimulated with insulin as described in methods. Total cell lysates were immunoprecipitated by using anti IR-alpha subunit antibody and ENPP1/IR interaction was evaluated by Western blot analysis with anti-ENPP1 specific antibody. Bars (upper panel) represent quantitative analysis of three independent Western blots (mean ± SEM), while a representative experiment is shown in the lower panel. As compared to HepG2 control cells, the amount of ENPP1 associated with IR was greatly increased in HepG2-K (*p = 0.002, second vs. first bars, n = 3 experiments in separate times) and even more profoundly in HepG2-Q (^§^p<0.001, third vs. first bars, n = 3 experiments in separate times) cells. When properly tested, a progressive increase of ENPP1/IR interaction was observed from control to HepG2-K and then HepG2-Q cells (p for trend<0.001).

Studies in non insulin target cells have suggested that ENPP1 inhibitory activity on IR autophosphorylation is mediated by ENPP1/IR interaction [24], [25]. Given our present data, we sought to replicate this finding also in typical insulin target cells, as is the case of HepG2. Upon insulin stimulation, as compared to control cells, the amount of ENPP1 associated with IR (Figure 2) was increased by 54 fold in HepG2-K and by 97 fold HepG2-Q cells. This confirms that also in liver cells ENPP1 inhibitory function on IR autophosphorylation is paralleled by ENPP1/IR interaction.

### Studies on insulin signaling in HepG2 and L6 cells

In order to characterize the effect of ENPP1 on downstream insulin signaling in typical insulin target cells, we investigated Akt-Ser^473^, GSK3-beta Ser^9^ and ERK1/2 Thr^202^/Tyr^204^ insulin-induced phosphorylation in HepG2 and L6 cells (Supporting Information S1). Briefly, transfection of *ENPP1*-K121 cDNA in both HepG2 and L6 cells was able to significantly reduce insulin-induced Akt-Ser^473^, GSK3-beta Ser^9^ and ERK1/2 Thr^202^/Tyr^204^ phosphorylation (Figures S2, S3, S4). In cells transfected with *ENPP1*-Q121 cDNA, this inhibitory effect was magnified in most of these steps (Supporting Information S1 and Figures S2, S3, S4). The general picture that can be drawn from these data strongly suggests that the greater inhibitory effect on IR autophosphorylation exerted by the Q121 variant is retained at most downstream post receptor steps, thus resulting in a more profound inhibition of the entire insulin signaling pathway in both cell types.

### Studies on insulin action in HepG2 and L6 cells

Insulin action on glucose metabolism was then assessed by studying mRNA level of the gluconeogenetic enzyme PEPCK in HepG2 cells and by investigating glucose transport and GLUT 4 expression level in L6 cells. PEPCK mRNA level was reduced by 65% upon insulin stimulation in untransfected HepG2 control cells. This reduction was progressively smaller in HepG2-K (54% reduction, p = 0.05 vs. reduction in control cells, n = 3) and in HepG2-Q cells (23% reduction, p = 0.009 vs. reduction in control cells, n = 3). Insulin stimulation induced a 60% increase of glucose uptake in untransfected L6 control cells. In contrast, insulin effect on glucose transport was totally abolished in L6-K (n = 3) and L6-Q cells (n = 3). Of note, as compared to control cells, GLUT 4 mRNA level was slightly and not significantly reduced in L6-K (13% reduction, p = 0.22, n = 3) and significantly decreased in L6-Q cells (25% reduction, p = 0.01, n = 3). Similarly, when GLUT 1 mRNA level was measured, no difference at all was observed in L6-K (n = 3), while a significant reduction was observed in L6-Q (26% reduction, p = 0.03, n = 3) as compared to control cells.

### Studies on beta-cell insulin secretion and survival

**Figure 3:**
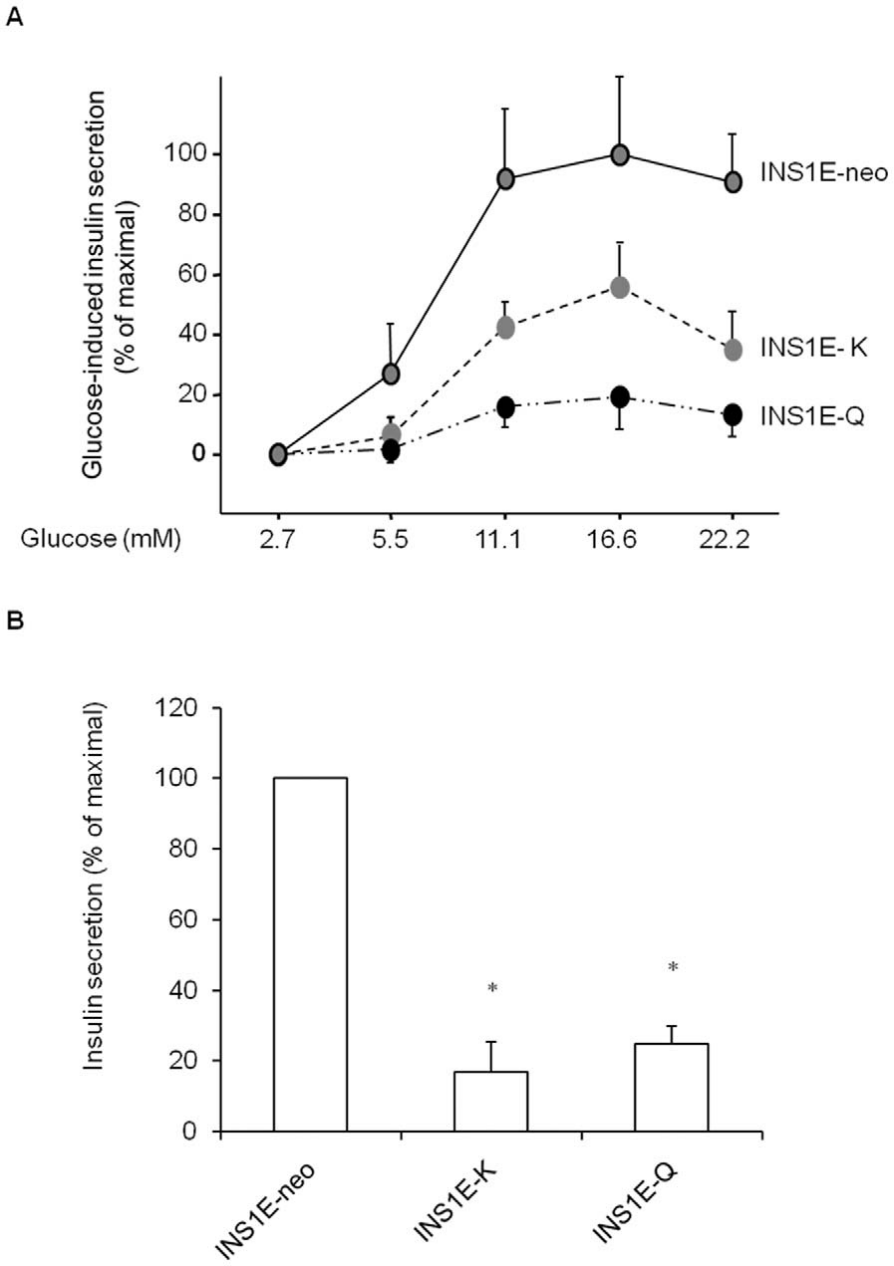
Studies on glucose-induced (A) and glyburide-induced (B) insulin secretion in INS1E cells. As compared to that of INS1E-neo control cells, glucose-induced insulin secretion *(* ***A*** *)* was markedly reduced in INS1E-K cells (p<0.005, n = 5 experiments in separate times) and almost completely abolished in INS1E-Q cells (p<0.0001, n = 5 experiments in separate times). Data are expressed as percentage of maximal secretion showed in INS1E-neo cells (mean ± SEM), which for glucose stimulation is obtained at 16.6 mM. When properly tested, a progressive reduction was observed from INS1E-neo to INS1E-K and then INS1E-Q cells (p for trend<0.001). As compared to that of INS1E-neo control cells, glyburide-induced insulin secretion *(* ***B*** *)* was almost completely abolished in both in INS1E-K and INS1E-Q cells (*p<0.0001 for both, n = 3 experiments in separate times). Data are expressed as percentage of glyburide-induced insulin secretion in INS1E-neo cells (mean ± SEM).

Increasing glucose concentration significantly stimulated insulin secretion in INS1E-neo control cells (Figure 3 A). This effect was markedly reduced in INS1E-K cells (60% reduction, Figure 3 A) and almost abolished in INS1E-Q cells (Figure 3 A). Glyburide significantly stimulated insulin secretion in INS1E-neo control cells (Figure 3 B); this effect was almost completely abolished in INS1E-K with no further effect observed in INS1E-Q cells (Figure 3 B).

**Figure 4:**
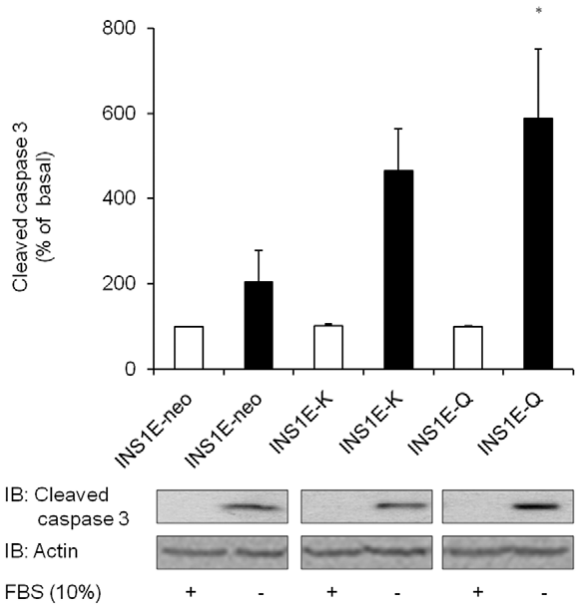
Apoptosis in INS1E cells. Apoptosis in INS1E was assessed by determining cleaved caspase-3 expression by Western blot. Equal amount of protein from cell lysates was separated by SDS-PAGE and probed with specific antibody. Bars (upper panel) represent quantitative analysis of cleaved caspase-3 calculated as percentage of that of non serum deficient control cells for each experiment (mean ± SEM), while representative immunoblot from the same experiment is shown in lower panel. A significant increment in cleaved caspase-3 expression was observed in INS1E-Q cells as compared to INS1E-neo control cells (*p<0.05, n = 4 experiments in separate times). When properly tested, a progressive increase was observed from INS1E-neo to INS1E-K and then INS1E-Q cells (p for trend = 0.038).

**Figure 5:**
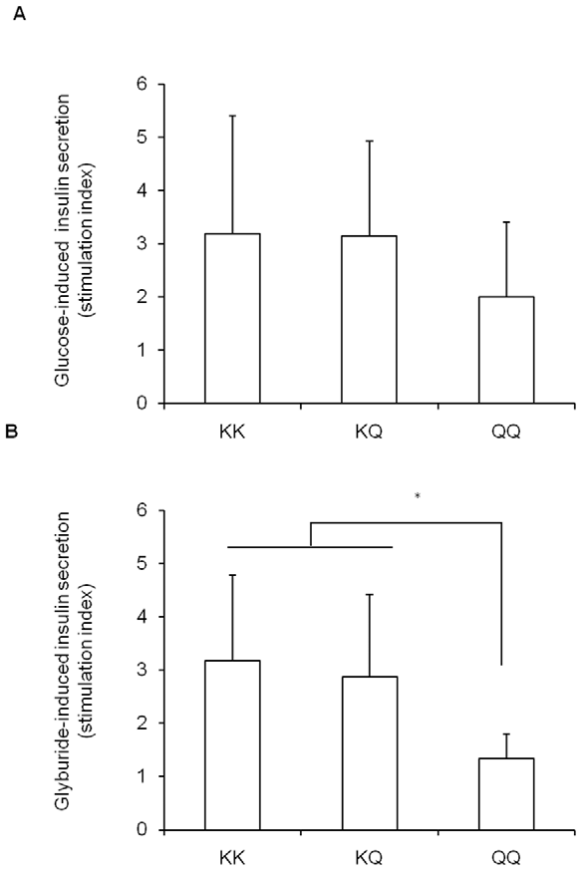
Insulin secretion in isolated human islets from 85 donors. Insulin secretion stimulated by glucose (16.7 mM, (***A***)) or glyburide (100 µM, (***B***)) was measured on isolated human islets as described in methods. Islets from QQ donors (n = 2) tended to secrete less insulin than islets from KK (n = 59) and KQ (n = 24) individuals under both conditions. Data (mean ± SEM) are expressed as stimulation index calculated by dividing stimulated insulin release (either by glucose 16.7 mM (***A***) or by glyburide 100 µM (***B***)) over basal insulin release (at glucose 3.3 mM).

We then tested the role of ENPP1 on INS1E beta-cell survival. Serum deficiency in INS1E-neo control cells induced a 2 fold increase of caspase-3 activation (Figure 4). This activation was exacerbated in INS1E-K cells (4.6 fold increase n = 4), and even more profoundly in INS1E-Q (5.9 fold increase, n = 4). The overall picture, that can be drawn by these findings, is that increasing ENPP1 expression in cultured beta-cells induces defective insulin secretion and increased apoptosis and that, also in this context, the Q121 variant is a gain of function aminoacid substitution. Based on our previous observation suggesting that homozygote individuals for the Q121 variant (i.e. QQ subjects) have defective insulin secretion [37], we sought to test the direct effect of this variant in the homozygote state also in isolated human islets from 85 donors. As shown in Figure 5, islets from the only two QQ donors, we had the chance to work with, tended to secrete approximately 50% less insulin under both glucose and glyburide stimulation.

## Discussion

An important finding of this study is that increasing ENPP1 expression in liver HepG2 and skeletal muscle L6 cells affects IR activation, downstream insulin signaling and insulin action on glucose metabolism. In both cell types, the relatively rare ENPP1 Q121 variant exhibits, as compared to the K121 variant, a greater inhibition of IR activation and of most subsequent insulin signaling and action steps, thus behaving as a gain of function aminoacid substitution. In HepG2 cells, the Q121 variant shows a stronger physical interaction with the IR. This strengthen the hypothesis emerged from previous studies carried out in non typical insulin target cells that, as compared to ENPP1 K121, the Q121 variant is a stronger inhibitor of insulin signaling and action because of a stronger protein-protein interaction with the IR [25].

An additional finding of this study, which is entirely novel, is that ENPP1 expression induces defective IR activation, reduced insulin secretion upon stimulation of both glucose and glyburide, and increased susceptibility to apoptosis in pancreatic beta-cells; also in most of these circumstances, the Q121 variant behaves as a gain of function aminoacid substitution. Although not conclusive because too sparse, also data in isolated human islets are in line with an inhibitory effect of the Q121 variant on insulin secretion, at least when present in the homozygous state.

Taken altogether, these findings clearly indicate that ENPP1, especially when the Q121 variant is operating, affects *in vitro* the most important tissues controlling glucose metabolism, including liver, skeletal muscle and insulin secreting beta-cells. A deleterious effect of ENPP1 on IR signaling and insulin action has been recently reported also in rat 3T3L-1 adipocytes [26], thus providing evidences for a role of ENPP1 in another very important insulin target tissue. These data in insulin target cells are fully compatible with those obtained in genetically modified animals in which changes in ENPP1 expression was directly correlated with deterioration of insulin sensitivity and abnormal glucose homeostasis [28], [29], [41].

As far as data on glucose- as well as glyburide-stimulated insulin secretion is concerned, our findings contribute to support an emerging scenario suggesting that IR signaling abnormalities have a direct, detrimental role on insulin secreting beta-cells [5], [6], [7], [19]. In this context, our present data are perfectly coherent with those reporting that other naturally occurring amino acid substitutions affecting insulin signaling, including IRS1 G972R [15], [17] and TRIB3 Q84R, directly affects insulin secreting beta-cells [8], [16]. The mechanism through which ENPP1 affects insulin secretion has not been addressed in this study. In beta-cells it has been reported that insulin signaling, through the activation of IRS1, PI3K [5], [42] and Akt-2 [43], increases Ca^++^ influx, especially from the endoplasmic reticulum and, therefore, facilitates insulin-containing granules trafficking and exocytosis. So, although entirely speculative, it can be hypothesized that, in cells over-expressing ENPP1, reduced insulin signaling causes defective intracellular Ca^++^ availability and eventually reduced glucose- as well as glyburide-stimulated insulin secretion.

As far as our present data on the Q121 variant is concerned, it is of note they are quite consistent with previous findings obtained *in vivo*. As a matter of fact, several [35], [36], [37] although not all [38] large studies have reported that individuals carrying the Q121 variant are insulin resistant as compared to KK subjects and that those carrying the QQ genotype also show defective insulin secretion [37]. Despite so many *in vitro* and *in vivo* findings coherently reported by several groups, the most updated meta-analysis, involving a huge number of individuals shows that a perfect proxy of the ENPP1 Q121 variant is not an established (i.e. at genome-wide level of statistical significance) marker of type 2 diabetes [44]. Several factors, intrinsic to study designs and data analyses of most GWAS (recently reviewed in [20]),might explain, at least partly, this apparent discrepancy. Nonetheless, the lack of established association with type 2 diabetes leaves uncertain the role of the Q121 variant on glucose homeostasis.

In conclusion, the data we obtained, clearly indicate that ENPP1 exerts *in vitro* a direct detrimental effect on the most important tissues for insulin sensitivity, insulin secretion and, eventually, glucose metabolism. Additional attempts, aimed at better clarify the role of ENPP1 as a pathogenic factor predisposing to insulin resistance-related abnormalities and type 2 diabetes, are definitively needed.

## Materials and Methods

### Cell Lines

HepG2 cells (ATCC, Manassas, USA) were maintained at 37°C and 5% CO_2_ in DMEM/F12 containing 10% FBS. L6 (ATCC, Manassas, USA) were maintained in DMEM containing 25 mM glucose with 10% FBS. Rat insulin-secreting INS1E cells (a kind gift from C. B. Wollheim, Department of Cell Physiology and Metabolism, University of Geneva, Geneva, Switzerland) were grown at 37°C and 5% CO_2_, in RPMI 1640 medium containing 2 mM L-glutamine supplemented with 10% heat-inactivated FBS.

### Plasmid

Full-length cDNA of *ENPP1*-K121 was kindly provided by Dr. I.D. Goldfine (San Francisco, University of California, USA) and the full-length cDNA of *ENPP1*-Q121 was generated by site directed mutagenesis as previously described [45]. Both cDNAs were cloned in mammalian expression vector pRK7.

### Transfections

HepG2 and L6 cells were transiently transfected with the full length cDNA of either *ENPP1*-K121 (HepG2-K and L6-K, respectively) or *ENPP1*-Q121 (HepG2-Q and L6-Q, respectively) by using TransIT reagent according to the manufacturers' instruction (Mirus), and then starved overnight in DMEM containing either 0.5% FBS or 0.25% BSA before experiments. ENPP1 protein expression in each condition was evaluated by Western blot analysis (Figure S1 A and S1 B). INS1E cells were either transfected with a plasmid (pRK7-neo) containing the neomycin resistance gene (INS1E-neo), or with the pRK7-neo plus the *ENPP1*-K121 cDNA (INS1E-K) or with the pRK7-neo plus *ENPP1*-Q121 cDNA (INS1E-Q) by using the Fugene Transfection Reagent (Roche, Germany) according to the company's instructions. Clones expressing a similar amount of ENPP1 were selected (Figure S1 C).

### Western blot

Cells lysates were separated by SDS-PAGE and transferred to nitrocellulose membrane (Amersham Pharmacia Biotech). Blots were probed with specific antibodies HRP-conjugated anti-goat, anti-mouse and anti-rabbit antibodies (Santa Cruz Biotechnology) and the chemiluminescent substrate (Super Signaling West Pico Thermo Scientific, Pierce or ECL by Amersham, GE Healthcare) was used for detection. Gel images were acquired by using Molecular Imager ChemiDoc XRS (Biorad) and analyzed by using Kodak Molecular Imaging Software 4.0 or IMAGEJ 1.40 g (Wayne Rasband, NIH).

### IR phosphorylation

HepG2, HepG2-K, HepG2-Q cells, L6, L6-K and L6-Q cells and INS1E-neo, INS1E-K and INS1E-Q cells were stimulated with 10^−6^ M insulin for 5 minutes at 37°C. Following cell lysis equal amount of protein was immunoprecipitated with anti-PY antibody (4G10 Platinum, Millipore, Italy), and analyzed by Western blot with anti IR-beta subunit antibody (C19, Santa Cruz Biotechnology, CA). IR phosphorylation was calculated as percentage of stimulated untransfected cells and expressed as means ± SEM.

### ENPP1/IR interaction

Following insulin stimulation (10^−6^ M for 5 minutes) and cell lysis, 2 mg of proteins were immunoprecipitated with anti IR alpha-subunit antibody and analyzed by Western blot analysis by using ENPP1 specific polyclonal antibody (N20 Santa Cruz Biotechnology, CA). Data were calculated as percentage of stimulated untransfected cells and expressed as mean ± SEM.

### Insulin downstream signaling

Following insulin stimulation (10^−6^ M for 5–10 minutes) and cell lysis, equal amount of protein was analyzed by western blot with the following specific antibodies: phospho-Akt Ser^473^, phospho-ERK1/2 Thr^202^/Tyr^204^, phospho-GSK3 beta Ser^9^ (Cell Signaling, Boston, MA). The blot were then stripped and re-probed with antibodies against Akt, ERK1/2 and GSK3 beta for normalization (Cell Signaling, Boston, MA). Data were calculated as percentage of stimulated untransfected cells and expressed as means ± SEM.

### PEPCK mRNA level

Thirty hours after transfection HepG2, HepG2-K and HepG2-Q cells were starved for 18 hours and then stimulated with 10^−6^ M insulin for 10 hours. Total RNA extraction and cDNA synthesis were performed as previously described [45]. PEPCK mRNA level was measured by RT-PCR by using the following primers: 5′-ATGTATGTCATCCCATTCAGC- 3′ and 5′-AATGTCATCACCCACACATTC-3′ [46]. Data were calculated as percentage of unstimulated untransfected HepG2 cells and expressed as means ± SEM.

### 2-Deoxy-D-glucose uptake in L6

2-Deoxy-D-glucose uptake in L6, L6-K and L6-Q cells was measured as previously reported [47]. Data were calculated as percentage over unstimulated cells and expressed as means ± SEM.

### GLUT 4 and GLUT 1 mRNA level

Total RNA from L6, L6-K and L-6Q cells was extracted and cDNA was obtained as previously described [45]. GLUT 4 and GLUT 1 mRNA level was measured by RT-PCR by using the following primers: 5′-CAGAAGGTGATTGAACAGAG-3′, 5′-AATGATGCCAATGAGAAAGG-3′ and 5′-CAGCCGATGTGACCCGAGAC-3′, 5′-GACGATACCCGAGCCGATGG-3′ respectively [48]. Data were calculated as percentage of untransfected L6 cells and expressed as means ± SEM.

### Insulin secretion in INS1E cells

Insulin secretion was evaluated as previously described [49]. Briefly, INS1E-neo, INS1E-K and INS1E-Q cells were seeded in six-well plates at a density of 8×10^5^ cells/well and, after 24 hours, the medium was removed and cells washed twice with glucose-free Krebs solution (pH 7.4). After a 60 minute preincubation period (37°C, 5% CO_2_) in Krebs solution containing 2.7 mM glucose, insulin secretion was determined in presence of increasing glucose concentrations (2.7; 5.5; 11.1; 16.6 and 22.2 mM) or 100 µM glyburide, a concentration used in previous *in vitro* studies [50]. After 60 minutes at 37°C, 5% CO_2_, aliquots of surnatant were taken for the measurement of insulin secretion (Rat/Mouse Insulin ELISA Kit, Billerica, MA, U.S.A.), while total protein content was determined using BCA protein Assay (Thermo scientific, Rockford, USA). Data were expressed as percentage of maximal secretion showed in INS1E-neo cells, which for glucose stimulation was obtained at 16.6 mM.

### Apoptosis assay

For caspase-3 experiments, INS1E-neo, INS1E-K and INS1E-Q cells were cultured in RPMI or starved overnight in serum-free RPMI containing 1.1 mM glucose and 0.1% BSA. After cells lysis, equal amount of protein was analyzed by Western blot, using cleaved caspase 3 (asp 175) antibody (Cell Signaling, Boston, MA, USA). Data were calculated as percentage of untreated INS1E-neo cells (% of basal) and expressed as means ± SEM.

### Pancreas donors

Pancreata were collected from 85 non diabetic brain-dead multiorgan donors (58±16 years 49.4% females) after informed consent was obtained in writing from family members, as previously reported [51]. The islet isolation centre has permission to prepare isolated islets and to use them for scientific research if they are not suitable for clinical islet transplantation, in accordance with national laws and our institutional ethical rules (Comitato Etico per la Sperimentazione dell'Azienda Ospadaliera Universitaria di Pisa).

### Human islet preparation

Pancreatic islets were prepared by collagenase digestion and density gradient purification, as previously reported in detail. After isolation, islets were cultured in M-199 culture medium as previously described [52].

### DNA extraction

DNA was extracted from batches of 2000 isolated islets in according to the Wizard genomic DNA purification protocol (Promega, Madison, WI, USA). Total DNA was quantified by absorbance at A260/A280 nm (ratio>1.65) in a Perkin-Elmer spectrophotometer, and its integrity assessed by electrophoresis on 1.0% agarose gel by ethidium bromide staining.

### Insulin secretion in isolated human islets

Insulin secretion was determined as previously described [52]. Briefly, following a 45 minute preincubation period at 3.3 mM glucose, batches of 15 islets of comparable size were kept at 37°C for 45 minutes in Krebs-Ringer bicarbonate solution (KRB) and 0.5% albumin, pH 7.4, containing 3.3 mM glucose. At the end of this period, the medium was completely removed and replaced with KRB containing 16.7 mM glucose and 3.3 mM G plus 100 µM glyburide [53], [54]. After an additional 45 minute of incubation, the medium was removed and stored at −20°C until insulin concentrations were measured by immunoradiometric assay (Pantec Forniture Biomediche, Turin, Italy). Data are expressed as stimulation index calculated by dividing stimulated insulin release (either by glucose 16.7 mM or by glyburide 100 µM) over basal insulin release (at glucose 3.3 mM).

### Genotyping of isolated human islets

Polymorphism K121Q (rs1044498) at *ENPP1* locus was genotyped by TaqMan allele discrimination (assay C_16190162_10, Applied Biosystems, Forster City, CA) on the HT7900 platform (Applied Biosystems). The failure rate was <1%. Genotyping quality was assessed by including positive controls with known genotypes. The agreement rate was >99%. Genotype distribution in the study sample was in Hardy-Weinberg equilibrium.

### Statistical analysis

Differences between mean values were evaluated by unpaired or paired Student's *t* test, as appropriate. General linear model was used to test the hypothesis that insulin signaling, action and secretion was progressively worse from control cells to K-cells and then Q-cells.

## Supporting Information

Figure S1 **ENPP1 expression in HepG2, L6 and INS1E cells.** HepG2 and L6 cells *(* ***A, B*** *)* were transiently transfected with the full length cDNAs of either *ENPP1*-K121 (HepG2-K and L6-K, respectively) or *ENPP1*-Q121 (HepG2-Q and L6-Q, respectively). ENPP1 protein content was then evaluated by Western blot analysis with specific antibody. Representative experiments are shown. INS1E cells *(* ***C*** *)* were stably transfected as described in methods. Two cell clones were isolated after transfection with a plasmid (pRK-7-neo) containing the neomycin resistance gene and co-transfected with *ENPP1*-K121 cDNA (INS1E-K, lane 3) or with *ENPP1*-Q121 cDNA (INS1E-Q, lane 4). Two clones expressing a similar amount of ENPP1 were selected. Three different experiments were carried out and a representative experiment is shown.
(TIF)

Figure S2 **Studies on Akt-S^473^ phosphorylation in HepG2 and L6 cells.** Cells were either transfected or not with *ENPP1*-K121 cDNA or with *ENPP1*-Q121 cDNA and stimulated with insulin as described in methods. Equal amount of protein from cell lysates was separated by SDS-PAGE and Akt-S^473^ phosphorylation was evaluated by using specific antibody by Western blot analysis. To evaluate Akt protein content, blots were stripped and reprobed with specific antibody. Bars (upper panels) represent quantitative analysis of Akt-S^473^ phosphorylation calculated in each single experiment as percentage of that of insulin stimulated untransfected cells (mean ± SEM), while representative immunoblots from the same experiment are shown in the lower panels. In HepG2 cells *(* ***A*** *)*, insulin strongly stimulated Akt-S^473^ phosphorylation. As compared to control cells, insulin effect was significantly and similarly reduced in both HepG2-K and HepG2-Q cells (*p<0.005, both fourth and sixth vs. second bar, n = 3 experiments in separate times). In L6 cells *(* ***B*** *)*, insulin strongly induced Akt-S^473^ phosphorylation. As compared to control cells, insulin effect was significantly reduced in both L6-K (*p = 0.02, fourth vs. second bar, n = 3 experiments in separate times) and L6-Q cells (^§^p<0.001, sixth vs. second bar, n = 3 experiments in separate times). When properly tested, a progressive reduction was observed from control to L6-K and then L6-Q cells (p for trend<0.001).
(TIF)

Figure S3 **Studies on GSK3-beta S^9^ phosphorylation in HepG2 and L6 cells.** Cells were either transfected or not with *ENPP1*-K121 cDNA or with *ENPP1*-Q121 cDNA and stimulated with insulin as described in methods. Equal amount of protein from cell lysates were separated by SDS-PAGE and GSK3-beta S^9^ phosphorylation was evaluated by using specific antibody by Western blot analysis. GSK3-beta protein content was evaluated by stripping and reprobing the same blot with specific antibody. Bars (upper panels) represent quantitative analysis of GSK3-beta S^9^ phosphorylation calculated in each single experiment as percentage of that of insulin stimulated untransfected cells (mean ± SEM), while representative immunoblots from the same experiment are shown in the lower panels. In HepG2 cells *(* ***A*** *)*, insulin greatly induced GSK3-beta S^9^ phosphorylation. As compared to control cells, this effect was reduced in HepG2-K (*p = 0.02, fourth vs. second bar, n = 3 experiments in separate times) and more strongly in HepG2-Q cells (^§^p<0.001, sixth vs. second bar, n = 3 experiments in separate times). When properly tested, a progressive reduction was observed from control to HepG2-K and then HepG2-Q cells (p for trend = 0.002). In L6 cells *(* ***B*** *)*, insulin induced GSK3-beta S^9^ phosphorylation. As compared to control cells, this effect was similarly reduced in L6-K (*p = 0.003, fourth vs. second bar, n = 3 experiments in separate times) and in L6-Q cells (^§^p<0.001, sixth vs. second bar, n = 3 experiments in separate times). When properly tested, a progressive reduction was observed from control to L6-K and then L6-Q cells (p for trend = 0.003).
(TIF)

Figure S4 **Studies on ERK1/2 Thr^202^/Tyr^204^ phosphorylation in HepG2 and L6 cells.** Cells were either transfected or not with *ENPP1*-K121 cDNA or with *ENPP1*-Q121 cDNA and stimulated with insulin as described in methods. Equal amount of protein from cell lysates was separated by SDS-PAGE and ERK1/2 Thr^202^/Tyr^204^ phosphorylation was evaluated by using specific antibody by Western blot analysis. ERK1/2 protein content was evaluated by stripping and reprobing the same blot with specific antibody. Bars (upper panels) represent quantitative analysis of ERK1/2 Thr^202^/Tyr^204^ phosphorylation calculated in each single experiment as percentage of that of insulin stimulated untransfected cells (mean ± SEM), while representative immunoblots from the same experiment are shown in the lower panels. In HepG2 cells *(* ***A*** *)*, insulin induced ERK1/2 activation. As compared to control cells, insulin effect was significantly reduced in both HepG2-K (*p = 0.003, fourth vs. second bar, n = 3 experiments in separate times) and slightly more HepG2-Q cells (^§^p<0.001, sixth vs. second bar, n = 3 experiments in separate times). When properly tested, a progressive reduction was observed from control to HepG2-K and then HepG2-Q cells (p for trend<0.001). In L6 cells *(* ***B*** *)*, insulin induced a great ERK1/2 activation. This effect was significantly reduced in L6-K (*p<0.001, fourth vs. second bar, n = 3 experiments in separate times) and more profoundly in L6-Q cells (*p<0.001, sixth vs. second bar, n = 3 experiments in separate times). When properly tested, a progressive reduction was observed from control to L6-K and then L6-Q cells (p for trend<0.001).
(TIF)

Supporting Information S1 Studies on insulin signaling in HepG2 and L6 cells.
(DOC)
