# Supplementary material for: ENPP1 Affects Insulin Action and Secretion: Evidences from In Vitro Studies
Source: PLoS One. 2011 May 5;6(5):e19462. doi: 10.1371/journal.pone.0019462 (PMC3088669; doi:10.1371/journal.pone.0019462)
Supplement: Supporting Information S1 — Studies on insulin signaling in HepG2 and L6 cells. (DOC) [file pone.0019462.s005.doc]

**Supporting Information**

*Role of ENPP1 Q121 variant on insulin downstream signaling*

**Akt-S^473^ phosphorylation**

As compared to control HepG2 cells, insulin stimulation of Akt-S^473^ phosphorylation was similarly reduced in HepG2-K and HepG2-Q cells (27-28% reduction, p < 0.005) (Figure S2 A).

As compared to control L6 cells, insulin stimulation of Akt-S^473^ phosphorylation was significantly reduced in L6-K (44% reduction, p = 0.015) and even more profoundly in L6-Q (75% reduction, p < 0.001) cells (Figure S2 B).

**GSK3-beta-S^9^ phosphorylation**

Insulin stimulation greatly induced GSK3-beta S^9^ phosphorylation in control HepG2 cells (Figure S3 A). This effect was reduced in HepG2-K (27% reduction, p = 0.02) and more strongly in HepG2-Q (46% reduction, p < 0.001) cells (Figure S3 A).

A similar pattern was observed in rat skeletal muscle cells with inhibition of insulin-stimulated GSK3-beta S^9^ phosphorylation being observed in L6-K (23% reduction, p = 0.003) and L6-Q (27% reduction, p < 0.001) as compared to control cells (Figure S3 B).

**ERK1/2 Thr^202^/Tyr^204^** **phosphorylation**

As compared to control HepG2 cells, insulin stimulation of ERK1/2 Thr^202^/Tyr^204^ phosphorylation was significantly reduced in HepG2-K (34% reduction, p = 0.003) and more profoundly in HepG2-Q (45% reduction, p < 0.001) cells (Figure S4 A).

Once again, a similar pattern was observed in L6 cells in which, as compared to control cells, insulin stimulation of ERK1/2 Thr^202^/Tyr^204^ phosphorylation was reduced in L6-K (37% reduction, p < 0.001) and more profoundly in L6-Q (57% reduction, p < 0.001) cells (Figure S4 B).
